# Immune-Related Multiple-Organs Injuries Following ICI Treatment With Tislelizumab in an Advanced Non-Small Cell Lung Cancer Patient: A Case Report

**DOI:** 10.3389/fonc.2021.664809

**Published:** 2021-09-02

**Authors:** Chao Deng, Meng Yang, Hong Jiang, Renbin Wang, Zhaojun Yang, Hongliang Sun, Huijuan Cui

**Affiliations:** ^1^Department of Medical Oncology, Integrated Traditional Chinese and Western Medicine, China-Japan Friendship Hospital, Beijing, China; ^2^Department of Respiratory and Critical Care Medicine, China-Japan Friendship Hospital, Beijing, China; ^3^Department of Cardiology, Integrated Traditional Chinese and Western Medicine, China-Japan Friendship Hospital, Beijing, China; ^4^Department of Neurology, China-Japan Friendship Hospital, Beijing, China; ^5^Department of Endocrinology, China-Japan Friendship Hospital, Beijing, China; ^6^Department of Radiology, China-Japan Friendship Hospital, Beijing, China

**Keywords:** immune checkpoint inhibitors (ICIs), tislelizumab, immune-related adverse events (irAEs), non-small cell lung cancer (NSCLC), programmed cell death-1(PD-1)

## Abstract

Immune-related adverse events (irAEs) following treatment with immune checkpoint inhibitors (ICIs) can affect almost any organ systems. Multiple-organs irAEs are a rare occurrence which makes its management and treatment very challenging. This is a case report of a 71-year-old man with advanced non-small cell lung cancer (NSCLC) who developed multiple-organs irAEs (lung, muscle, myocardium, liver, and pituitary) after a single cycle (21 days) of the BGB-A317 (Tislelizumab). After more than two months of immunosuppression treatment with glucocorticoids, the tumor and inflammatory lesions in the lung were reduced. The levels of serum creatase, cardiac troponin T (TNT), and hepatic transaminase were also reduced. Four months after the termination of ICI therapy, the lung tumor reappeared in the previous site. This rare case report supplies several experiences in the management of multiple-organs irAEs, including full-scale monitoring of immunological indicators, early differential diagnosis, and prompt glucocorticoid therapy. This patient was not a candidate for the ICI re-challenge therapy due to the number and seriousness of irAEs. Multiple-organs irAEs add complexity to the management, and additional research is needed to develop optimal therapeutic guidelines.

## Introduction

Immune checkpoint inhibitors (ICIs) have emerged as revolutionary and promising immune-based therapies for cancer, demonstrating durable antitumor responses in multiple cancer types (1, 2). However, T cells can be activated by ICIs, resulting in immune-related adverse events (irAEs), which can affect multiple body systems, primarily the pulmonary, endocrine, skin, and gastrointestinal systems (3, 4).

The occurrence of irAEs has been associated with improved tumor responses and survival outcomes in most cancer patients undergoing ICI therapy (5, 6). The number of irAEs is related to the antitumor effects of ICIs used as well as to the degree of autoimmune activation by the ICIs (7, 8).

A single target organ is most often affected in mild irAEs, which can occur in 60%–70% of patients accepting a monotherapy of programmed cell death-1 (PD-1)/programmed cell death-Ligand 1 (PD-L1) inhibitor (9). However, both single and multiple-organs irAEs can be life-threatening. The myositis occurred in approximately 0.6% of ICI-treated patients, however, among the myositis cases, 95.3% are serious to require at least a hospitalization, with a fatality rate of 22.3% (10). The fatal outcome may be variably impacted due to the other concomitant irAEs, such as myasthenia gravis, rhabdomyolysis, and myocarditis (11, 12). The incidence of ICI-associated myocarditis has been reported to range from 0.06% to 1%. It is difficult to diagnose for lack of specificity in the clinical presentation compared to other cardiovascular diseases (13). Hypophysitis is also rare, with an incidence of only 0.4% for PD-1 inhibitors (14). Pneumonitis and hepatitis are observed much more frequently, which occurs in 3%–10% and 1%–10% of patients accepting ICI, respectively (15, 16).

Tislelizumab is an anti-PD-1 monoclonal antibody, that is similar to Nivolumab and Pembrolizumab in anti-tumor efficacy, safety, and tolerability for advanced NSCLC patients.

We report a case of a 71-year-old man with advanced non-small cell lung cancer (NSCLC) who developed successive multiple-organ irAEs including myositis, myocarditis, pneumonia, hepatitis, and hypophysitis, after the first cycle treatment with Tislelizumab. The tumor in the lung nearly disappeared. IrAEs were reduced after the discontinuation of PD-1 inhibitor and the initiation of treatment with corticosteroids. Unfortunately, the lung tumor reoccurred in the same site after termination of ICI therapy but was reduced with subsequent chemotherapy.

## Case Presentation

This case report involved a 71-year-old male with advanced NSCLC (cT2N2M0 IIIa), without tumor driver genes mutations. The patient was diagnosed by percutaneous needle lung biopsy (PNLB) in October 2019. The main past history including the anticoagulant therapy for thrombus in the lower extremity veins from December 2019, a smoking history of 50 years, and the death of his sister from ovarian cancer. The Tumor Mutational Burden (TMB) of the patient was 9.68 mut/Mb. The expression rate of PD-L1 was 80% to 90% in tumor cells, and approximately 1% in immune cells. The patient was treated with first-line chemotherapy alone (pemetrexed plus carboplatin), rather than a combination therapy with ICIs or bevacizumab, due to medical expense and anticoagulant therapy (for thrombus in the lower extremity veins). When the tumor did not respond to this treatment, the patient agreed to a treatment of a single cycle of the ICI Tislelizumab (200 mg d1, 21 days a cycle; BeiGene, China) on March 12, 2020. Fever, weakness, and cough appeared in the afternoon and evening of the first day of treatment.

**Figure 1:**
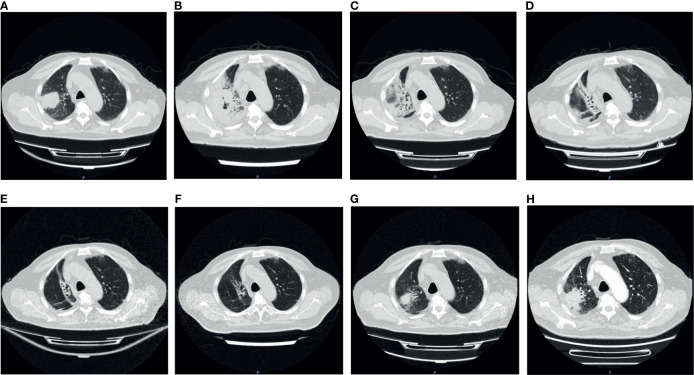
The variation of the tumor and inflammation in Chest CT. **(A)** Pre-immunotherapy: the tumor was seen in the right lung; **(B)** 2 weeks after immunotherapy: patchy shadows appeared around the tumor and air holes occurred in the tumor; **(C)** 4 weeks after immunotherapy: more air holes developed in the tumor and patchy shadows; **(D)** 5 weeks after immunotherapy: the tumor and patchy shadows were dissipating; **(E, F)** 8/12 weeks after immunotherapy: the tumor disappeared, with several linear shadows leaving; **(G)** 16 weeks after immunotherapy: the tumor reappeared in original site; **(H)** 18 weeks after immunotherapy: the tumor enlarged.

**Figure 2:**
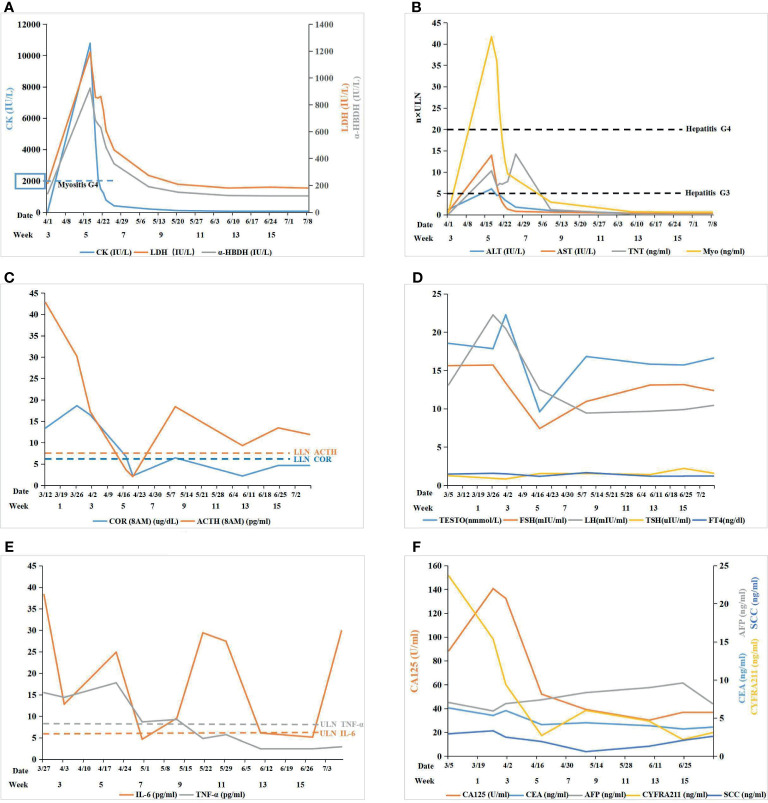
**(A)** Myositis: the level of serum creatase was used to monitor immune related myositis after immunotherapy; **(B)** Myocarditis+ Hepatitis: cardiac markers and hepatic transaminase were used to monitor myocarditis and hepatitis, respectively, after immunotherapy; **(C)** Pituitary-Adrenal Axis: the level of COR and ACTH were used to monitor the function of the pituitary-adrenal axis: COR and ACTH both declined remarkably after ICI therapy; ACTH was maintained at a low level while COR was still lower than the normal value by glucocorticoid replacement therapy; **(D)** Pituitary-Gonad Axis: the level of FSH, LH, and TESTO were used to monitor the function of the pituitary-gonad axis: all of them fluctuated in the range of normal values; TSH and FT4 were both normal in the pituitary-thyroid axis; **(E)** Autoimmune Reponse: IL-6 and TNF-α was used to monitor the autoimmune response induce by ICI: IL-6 fluctuated beyond the upper limit of normal (ULN) while TNF-α fell to normal gradually after glucocorticoid replacement therapy; **(F)** Tumor Marker: tumor markers was used to monitor efficacy of ICI.

A computerized tomography (CT) of chest scan showed the presence of the tumor before ICI treatment (**Figure 1A**), and two weeks after the treatment, interstitial pneumonia appeared around the tumor (**Figure 1B**). An increase in serum interleukin-6 (IL-6) and tumor necrosis factor (TNF) was detected (**Figure 2E**). Myalgia occurred 10 days after the termination of ICI treatment. Anti-inflammatory treatment (Prednisone, 20 mg, qd; meloxicam,7.5 mg, qd) was administrated. Five days later, the patient felt weakness in the lower extremities (muscle force, grade 3) and could not stand or walk. The patient would gasp for breath after activity. It is not uncommon that ICI treatment of patients can result in the late-onset of immunological complications, including those involving the musculature, nervous, pulmonary, and endocrine systems.

**Figure 3:**
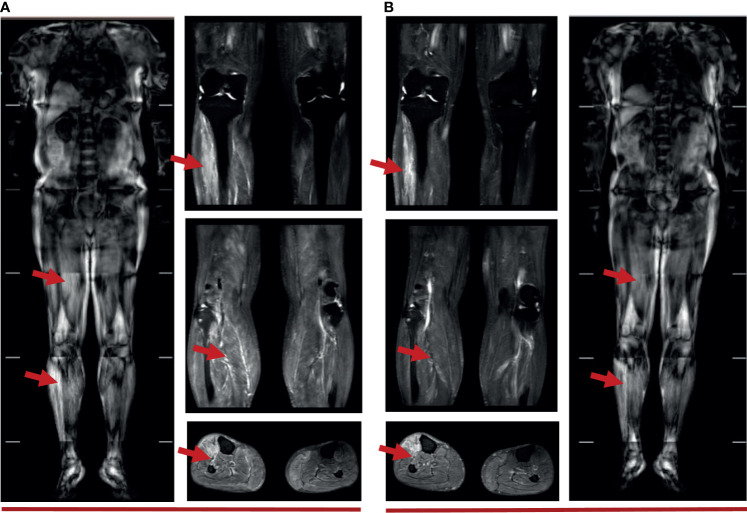
**(A)** Muscle MRI of 5 weeks after immunotherapy: diffuse strip-shaped high-signal shadows were seen in the back and lower limbs, as indicated by the red arrows; **(B)** Muscle MRI of 10 weeks after immunotherapy: high-signal shadows receded after 40 days of glucocorticoid treatment, especially in the right lower limbs, as indicated by the red arrows.

The ability of the patient to perform daily physical activities was limited, especially in the lower limbs. Serum levels of creatine kinase (CK), lactic dehydrogenase (LDH), and α-hydroxybutyric dehydrogenase (α-HBDH) had increased (**Figure 2A**). Electromyography showed neural normal conduction, myotonic discharges in the bilateral anterior tibial, right quadriceps, iliopsoas, and biceps brachii were detected. Magnetic resonance imaging (MRI) found a diffuse exudation in the muscles of the backside and lower limbs (**Figure 3A**). Although the antinuclear antibody spectrum, myositis auto-antibody spectrum, immunoglobulin, and alexin were all negative, severe myositis was still considered a possible irAE based on the clinical manifestation above.

Myocarditis was believed to be another irAE concurrent with myositis. The level of cardiac troponin T (TNT) was remarkably high (**Figure 2B**). An electrocardiogram (ECG) indicated complete right bundle branch block (CRBBB), and potential inferior myocardial infarction, rather than the normal manifestation prior to ICI therapy (**Supplementary Image 1**). Cardiac ultrasonography (UCG) and cardiac magnetic resonance (CMR) did not indicate abnormalities in the structure and function of the heart. The possibility of acute myocardial infarction (AMI) and pulmonary embolism (PE) were excluded by coronary and pulmonary angiography. Myocardial damage was considered to be an irAE induced by ICI therapy based on the clinical manifestations above.

Both serum glutamic-pyruvic transaminase (ALT) and glutamic-oxaloacetic transaminase (AST) also increased (**Figure 2B**). The patient denied a history of hepatic diseases. Hepatic damage was considered an irAE based on a comparison of AST and ALT pre- and post-ICI treatment.

The level of cortisol (COR) and adrenocorticotrophic hormone (ACTH) was lower than normal values from April 17, which fluctuated following glucocorticoid therapy (**Figure 2C**). The levels of follicle-stimulating hormone (FSH), luteinizing hormone (LH), and testosterone (TESTO) were normal, yet transitorily lower than the base value on April 17, while thyroid-stimulating hormone (TSH) and free thyroxine (FT4) were normal. (**Figure 2D**). Secondary adrenal insufficiency was clinically diagnosed as an irAE (hypophysitis) with isolated ACTH deficiency (IAD) despite the lack of a pituitary MRI.

**Figure 4:**
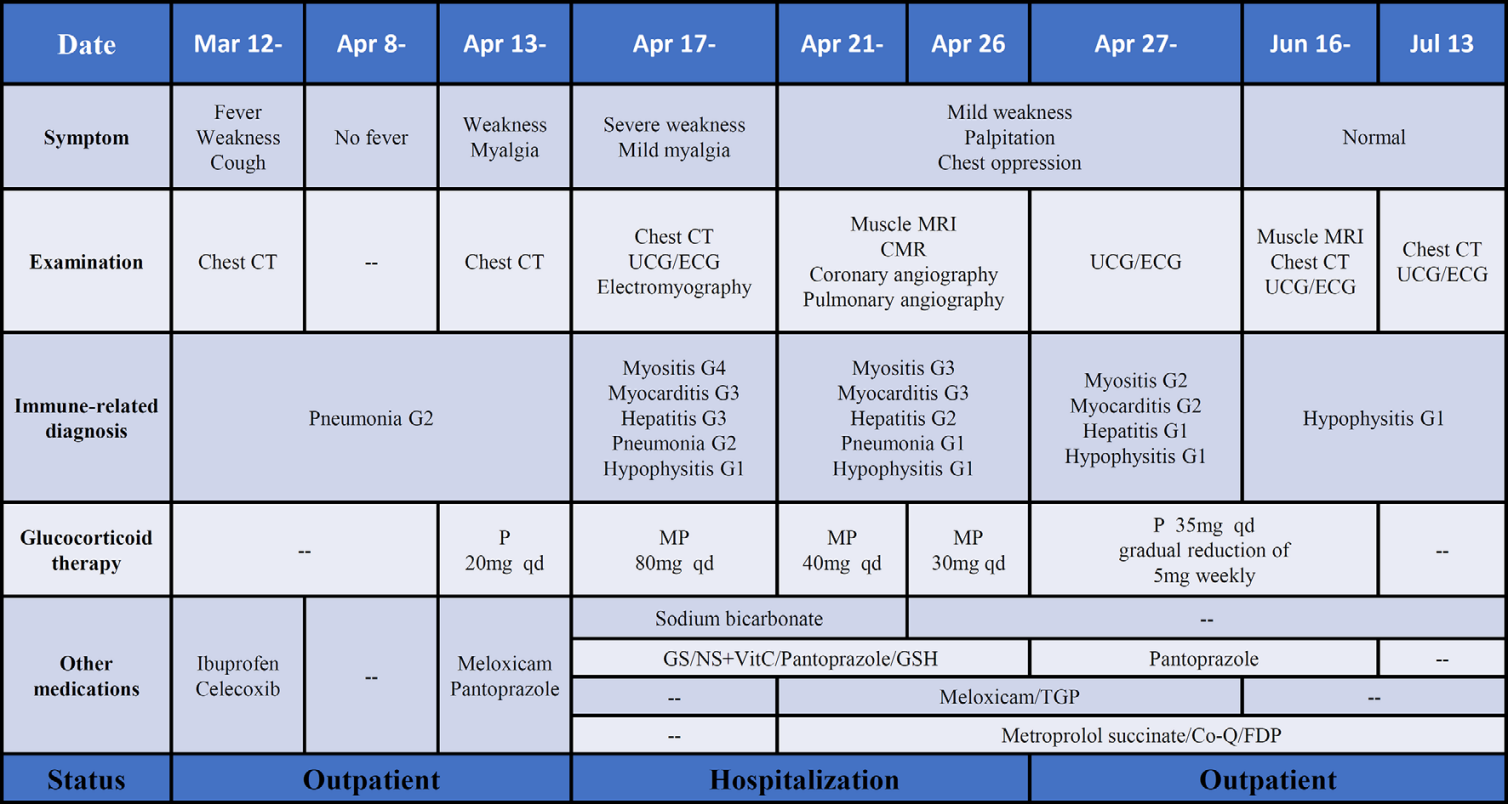
The process of diagnosis and treatment of irAEs after ICI therapy. P, prednisone; MP, methylprednisolone; GS, glucose solution; NS, normal saline; TGP, total glucosides of paeonia; Co-Q, coenzyme Q; FDP, fructose1, 6-diphosphate. Note: The grade of irAEs refer to the management of immunotherapy-related toxicities from the NCCN clinical practice guidelines and the consensus recommendations from the Society for Immunotherapy of Cancer (SITC) Toxicity Management Working Group.

This patient initially accepted 80 mg of methylprednisolone intravenously to suppress the autoimmune reaction with the dose reduced gradually, until adjusted to oral prednisone with reduction sequentially. In addition, meloxicam (7.5 mg, qd) and total glucosides of paeonia (TGP) (0.6 g, tid) were administered to suppress inflammation and to regulate immune function. Coenzyme Q (Co-Q), fructose1, 6-diphosphate (FDP), and vitamin C were administered to protect the myocardium. Metroprolol succinate was administered to alleviate the workload of heart. To reverse the ICI-induced hepatic damage, reduced glutathione was administered. After the comprehensive treatment, the abnormal indications induced by ICI gradually recovered to normal. And the diffuse exudation reduced in the muscles of the right lower limbs (**Figure 3B**). The therapeutic process of irAEs is shown in **Figure 4**.

Although the multiple-organs irAEs appeared, the efficacy of ICI therapy was encouraging. Following the treatment, the tumor lesion and inflammation around the tumor both diminished gradually until the final disappearance (**Figures 1C–F**). Meanwhile, the serum tumor markers (CA125, CYFRA211, and CEA) showed a downward trend (**Figure 2F**). Despite this initial success, cancer reoccurred in the right lung 4 months after the termination of ICI treatment (**Figures 1G, H**).

Positron emission tomography-computed tomography (PET-CT) examination confirmed the relapse of the tumor (**Supplementary Image 2A**). As a result of the seriousness of the irAEs of the patient, an ICI re-challenge therapy was not administered, and chemotherapy (vinorelbine plus carboplatin) plus bevacizumab was initiated as the subsequent treatment. After two cycles of the treatment, the area of the right lung lesion was reduced (**Supplementary Image 2B**).

After six cycles of the chemotherapy (vinorelbine plus carboplatin) plus bevacizumab, the right lung lesion increased again and directly invaded the pleura, then the patient chose the argon-helium knife cryotherapy as local therapy, and the chemotherapy (Abraxane plus carboplatin) plus bevacizumab as systemic therapy. However, after two cycles of the chemotherapy, serious myelosuppression appeared, and, meanwhile, the physical condition was poor, the patient began to accept the optimal supportive care until August 8, 2021.

The overall survival of this patient is 22 months up to now. Although multiple-organs irAEs occurred after only one cycle of immunotherapy, the patient may still obtain a benefit from the remarkable efficacy of immunotherapy to prolong survival.

## Discussion

As far as we know, this is a rare case report that describes the development of multiple-organs irAEs after a single cycle of ICI monotherapy (Tislelizumab) in a 71-year-old man treated for NSCLC. After ICI therapy, pneumonitis appeared before an acute onset of myositis, with the subsequent and concomitant irAEs of myocarditis, hepatitis, and hypophysitis.

Myositis is an ICI-induced neuromuscular irAE, with an all-grade incidence of less than 1% (17). Our reported case of ICI-related myositis is consistent with previous cases, in which muscle weakness of the limbs (32%), myalgia (42%), and CK elevation (43%) are manifested. As our observations, myositis-associated auto-antibodies are not detected in most cases (17). Electromyography, muscle MRI, and muscle biopsy are needed for the diagnosis of myositis. A muscle biopsy of this case was not performed because of the risk of bleeding and poor healing resulting from anticoagulant therapy used for thrombus in the lower extremity veins.

Myocarditis is the most fatal complication of ICI therapy with a mortality of 50% (18), which can occur concomitantly with other irAEs, such as myositis (17.3%), hepatitis (6.8%), and pneumonitis (4.5%) (19). The clinical manifestation of myocarditis can range from mild, nonspecific symptoms to sudden cardiac death, and may present with the decline of left ventricular ejection fraction (EF) and arrhythmia in fulminant progression (20, 21). The patient did not display specific cardiac symptoms but had high levels of TNT along with abnormal electrical conduction of cardiac rhythms. However, the normal findings on both echocardiogram and CMR do not rule out myocarditis (22). As a gold standard for diagnosis, endomyocardial biopsy is limited due to its invasive nature. Thus, it is recommended that broad differential diagnoses by a cardiologist be considered for patients with suspected myocarditis.

Pneumonitis, if not treated, is a life-threatening irAE, accounting for 28% of ICI-induced deaths (23). The risk of pulmonary toxicity occurs earlier and is more extensive in NSCLC than in other tumor types (24). The chest CT of this patient showed a large shadow around the tumor lesion prior to the appearance of subsequent irAEs. In addition to the lung, liver and endocrine are also the common organ sites in multiple-organs irAEs reported in a review (25). Concerning liver and pituitary function, we detected no symptoms in the patient beyond the elevation of ALT/AST levels and the decline of COR/ACTH levels. Secondary adrenal insufficiency due to hypophysitis was diagnosed based on the detection of low cortisol levels. Normal secretion of pituitary hormones other than ACTH is termed isolated ACTH deficiency (IAD), a rare pituitary disorder in which structural pituitary defects are absent typically (26), which is similar to the mild pituitary enlargement in most ICI-related hypophysitis (27).

The mechanism by which multiple-organs irAEs manifest is still poorly understood. It is possible that common antigens or antibody receptors coexist in the affected organs, and that certain antigens are either released from tumor cells killed by T lymphocytes or shared between tumor and normal tissues, resulting in uncontrolled autoimmune reactions across multiple organ systems (20, 28).

Guidelines have been established for the management and treatment of individual organ irAEs (29), but there is little experience in treating multiple-organs irAEs. It is important to seek consultation from multiple specialists for the differential diagnoses of non-immune diseases versus ICI-induced irAEs. Meanwhile, the appropriate monitoring is needed in the balance between the efficacy and safety of the ICI therapy. The correlation between increased IL-6 and grade 3 or greater irAEs was identified in a retrospective analysis (30), IL-6 has been reported to be a biomarker in autoimmune responses in a preliminary study (31), TNF-α was another potential biomarker of irAEs in a plasma biomarkers screening (32). In our case, the level of IL-6 was markedly elevated when irAEs occurred, and after glucocorticoid therapy, IL-6 appeared to temporary decline, but it still fluctuated beyond the upper limit of normal in the whole treatment, probably because of the degree of autoimmune responses and the gradual reduction of glucocorticoid. Thus, the potential value of IL-6 as a biomarker still requires further investigation. While, TNF-α fell to normal gradually after glucocorticoid replacement therapy, which was almost consistent with the previous studies.

A key treatment in this report was the early application of low-dose steroids with dose adjustment by the evolution and severity of multiple-organs irAEs. In our patient, low-dose prednisone was administrated with the initial occurrence of myalgia, which may be beneficial to the suppression of the fulminant progress of multiple-organs irAEs, especially for the fatal complication, such as myocarditis and myositis. This point still requires more evidences to support.

ICI re-challenge therapy after the development of irAEs is still in dispute. In a cross-sectional cohort study, the recurrence rate of the same irAE was 28.8% with re-challenge using the same ICI after discontinuation of ICI therapy (33). Patients with grade 3 or 4 irAEs tended to develop severe irAEs on re-challenge with an ICI (34). Because of the seriousness of irAEs and the short-lived response to tumor occurring in this patient, ICI re-challenge therapy was not considered as the next treatment.

As a sort of ICI used in our case, Tislelizumab is an anti-PD-1 monoclonal antibody, with a different binding orientation to PD-1 in comparison with other PD-1 inhibitors such as pembrolizumab and nivolumab (35). The clinical evidence for Tislelizumab is limited at present, though it has demonstrated encouraging results across several clinical trials for the treatment of advanced NSCLC (36). As shown in **Supplementary Table 1** (37–41), Tislelizumab monotherapy is similar to Nivolumab and Pembrolizumab in anti-tumor efficacy, safety, and tolerability, but more clinical data are still needed to feature Tislelizumab.

## Conclusion

Our case report supplies several experiences in the management of multiple-organs irAEs, including full-scale monitoring of immunological indicators, early differential diagnosis, and prompt glucocorticoid therapy, which are crucial for the outcome of patients with multiple-irAEs, especially for the deadly complication like myocarditis.

## Data Availability Statement

The original contributions presented in the study are included in the article/**Supplementary Material**. Further inquiries can be directed to the corresponding author.

## Ethics Statement

Written informed consent was obtained from the individual(s) for the publication of any potentially identifiable images or data included in this article.

## Author Contributions

CD is the drafter of the manuscript. HC proposed the concept of this case report. HC and CD administered the whole course of diagnosis and treatment in this patient. HC, MY, HJ, RW, and ZY contributed to the multi-disciplinary consultation in immune-related pneumonia, myocarditis, myositis, hepatitis, and hypophysitis, respectively. HS was responsible for radiological imaging diagnosis in CT and MRI. All authors contributed to the article and approved the submitted version.

## Conflict of Interest

The authors declare that the research was conducted in the absence of any commercial or financial relationships that could be construed as a potential conflict of interest.

## Publisher’s Note

All claims expressed in this article are solely those of the authors and do not necessarily represent those of their affiliated organizations, or those of the publisher, the editors and the reviewers. Any product that may be evaluated in this article, or claim that may be made by its manufacturer, is not guaranteed or endorsed by the publisher.

## Supplementary Material

Supplementary Image 1 The baseline and abnormal ECG after immunotherapy. **(A)** Pre-immunotherapy: normal; **(B)** 5 weeks after immunotherapy: sinus tachycardia, CRBBB, and potential inferior myocardial infarction.

Supplementary Image 2 The image change of the lung before and after the subsequent anti-cancer treatment following irAEs. **(A)** PET-CT before treatment: tumor with hypermetabolism was seen in the right lung; **(B)** Chest CT after two cycles of treatment: the tumor was reduced with air holes formed.

Supplementary Table 1 Different PD-1 inhibitors monotherapy trials in patients with advanced NSCLC.
