# Supplementary figures and images for: Immune-Related Multiple-Organs Injuries Following ICI Treatment With Tislelizumab in an Advanced Non-Small Cell Lung Cancer Patient: A Case Report
Source: Front Oncol. 2021 Sep 2;11:664809. doi: 10.3389/fonc.2021.664809 (PMC8443792; doi:10.3389/fonc.2021.664809)

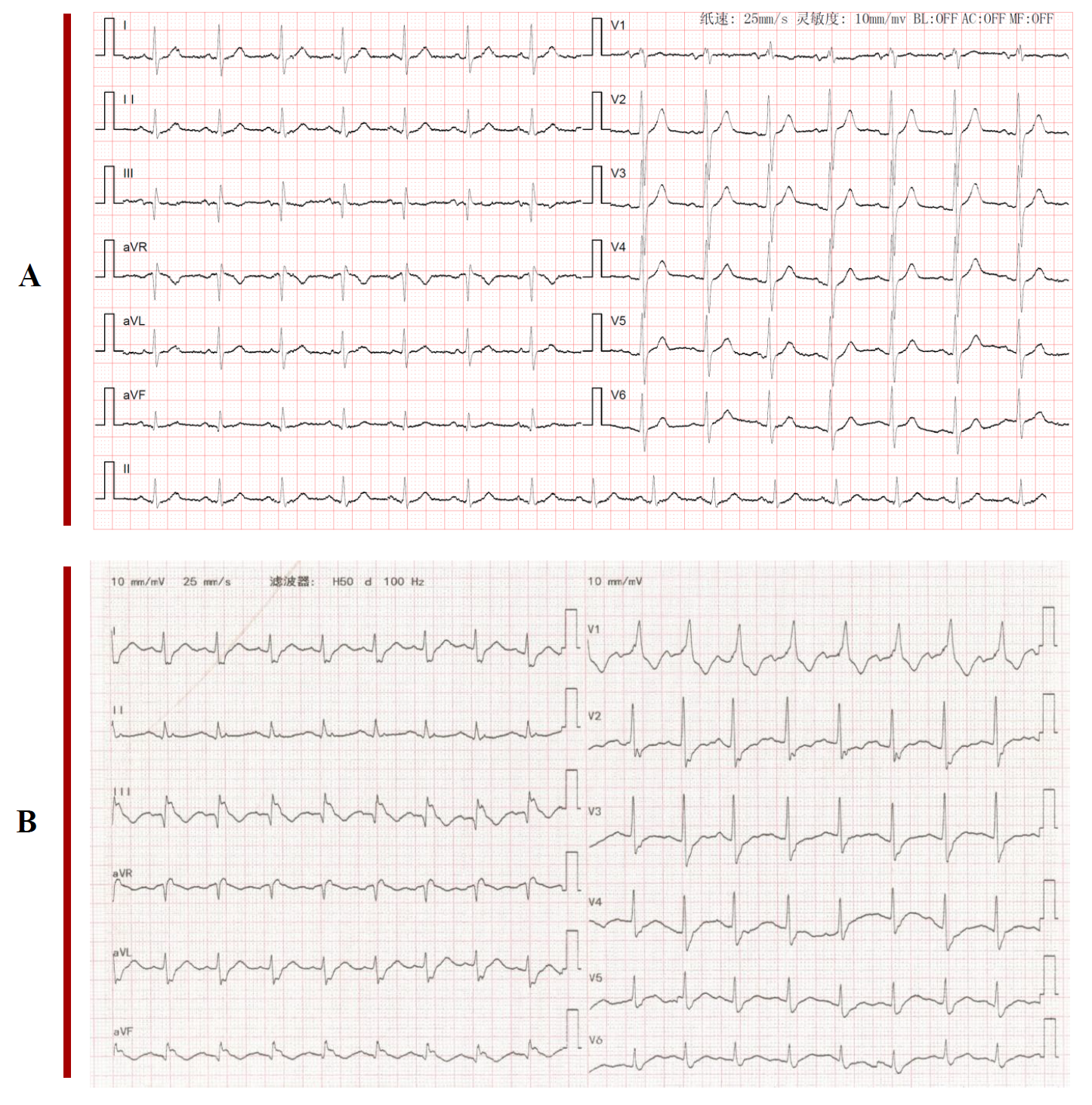

Supplement: Supplementary Image 1 — The baseline and abnormal ECG after immunotherapy. (A) Pre-immunotherapy: normal; (B) 5 weeks after immunotherapy: sinus tachycardia, CRBBB, and potential inferior myocardial infarction. [file Image_1.tiff]

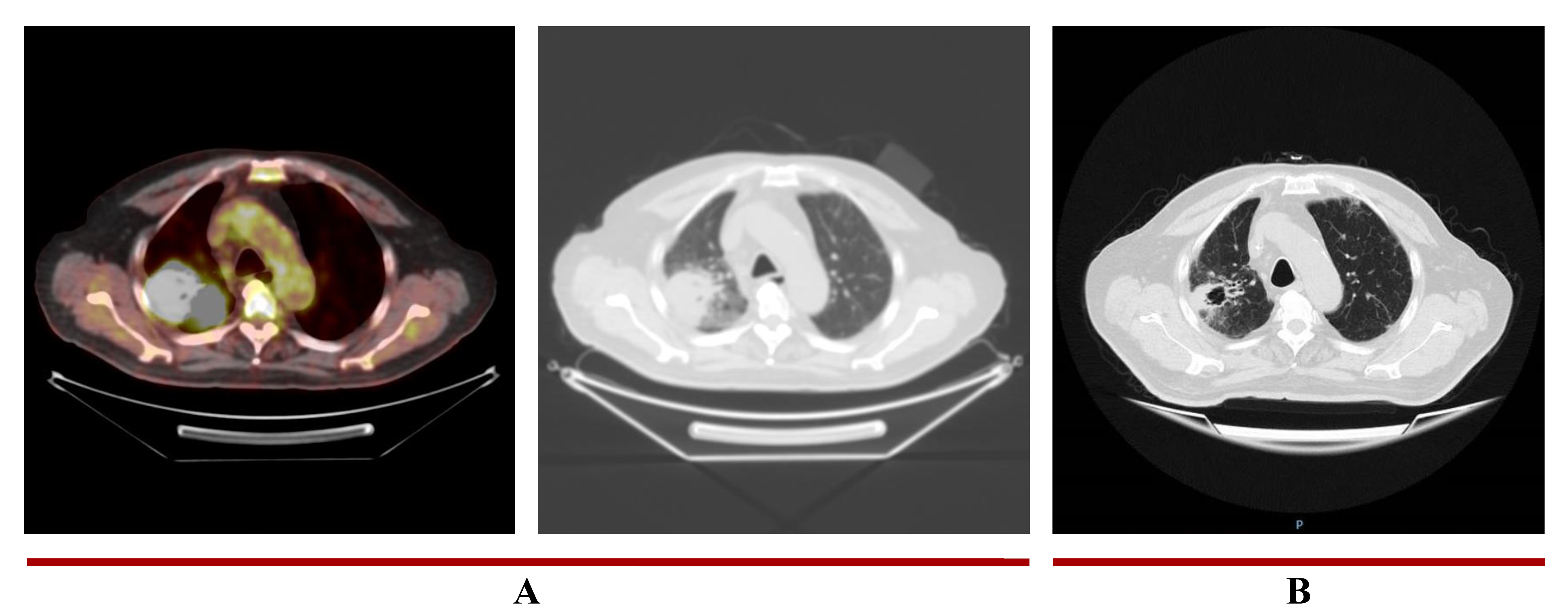

Supplement: Supplementary Image 2 — The image change of the lung before and after the subsequent anti-cancer treatment following irAEs. (A) PET-CT before treatment: tumor with hypermetabolism was seen in the right lung; (B) Chest CT after two cycles of treatment: the tumor was reduced with air holes formed. [file Image_2.tiff]
