# Supplementary material for: Immune-Related Multiple-Organs Injuries Following ICI Treatment With Tislelizumab in an Advanced Non-Small Cell Lung Cancer Patient: A Case Report
Source: Front Oncol. 2021 Sep 2;11:664809. doi: 10.3389/fonc.2021.664809 (PMC8443792; doi:10.3389/fonc.2021.664809)
Supplement: Supplementary Table 1 — Different PD-1 inhibitors monotherapy trials in patients with advanced NSCLC. [file Table_1.docx]

**Supplementary Table 1 Different PD-1 inhibitors monotherapy trials in patients with advanced NSCLC**

| **ICI** |  | **Tislelizumab** |  | **Nivolumab** | |  | **Pembrolizumab** | |
| --- | --- | --- | --- | --- | --- | --- | --- | --- |
| **Trial** |  | **BGB-A317-102 (37)** |  | **CheckMate 026 (38)** | **CheckMate 078 (39)** |  | **KEYNOTE-024 (40)** | **KEYNOTE-042**  **(41)** |
| Phase |  | 1/2 |  | 3 | 3 |  | 3 | 3 |
| Comparator |  | None |  | Plat | Docetaxel |  | Plat | Plat |
| N |  | 300 |  | 423 | 504 |  | 305 | 1274 |
| Patient population |  | Advanced solid tumors with measurable disease |  | First-line stage IV or recurrent NSCLC; PD-L1 ≥5% | Advanced NSCLC after progression on plat |  | First-line advanced NSCLC; PD-L1 ≥50% | First-line advanced or metastatic NSCLC; PD-L1 ≥1% |
| **Efficacy** | | | | | | | | |
| ORR (%) |  | 18^a^ |  | 26 | 17 |  | 45 | PD-L1≥50%/20%/1%  39/33/27 |
| mOS (month) |  | NR^a^ |  | 14.4 | 12 |  | NR | PD-L1≥50%/20%/1%  20.0/17.7/16.7 |
| mPFS ( month ) |  | 4^a^ |  | 4.2 | 2.8 |  | 10.3 | PD-L1≥50%/20%/1%  7.1/6.2/5.4 |
| **AEs** | | | | | | | | |
| All Grade (%) |  | 57 |  | 71 | 64 |  | 76.7 | 63 |
| Grade ≥ 3 (%) |  | 11 |  | 18 | 10 |  | 31.2 | 18 |
| Led to treatment  discontinuation (%) |  | 8 |  | 10 | 4 |  | 13.6 | 9 |

**^a^** only data for the efficacy of Tislelizumab.in NSCLC are presented

**Abbreviations:** Plat: platinum-based chemotherapy; ORR: objective response rate; mOS: median overall survival; NR: not reached; mPFS, median progression-free survival
